# Breaking barriers and saving lives: overcoming stigmas and enhancing childhood cancer awareness in South Africa

**DOI:** 10.3332/ecancer.2025.1977

**Published:** 2025-08-29

**Authors:** Lauren Pretorius, Adri Ludick

**Affiliations:** 1 Chief Executive Officer, Campaigning for Cancer NPC, Craighall, Johannesburg 2196, South Africa; 2 Programme Development Manager, CHOC Childhood Cancer Foundation, Rivonia, Johannesburg 2128, South Africa

**Keywords:** childhood cancer, stigma, myths, South Africa, civil Society, advocacy, traditional health practitioner

## Abstract

There is a critical need for overcoming stigmas and enhancing awareness of childhood cancer in South Africa, where the underreporting and late diagnosis of cases significantly impact survival rates. Despite the global increase in childhood cancer cases, South Africa's statistics reveal a stark contrast, with only 70–80 children diagnosed per million, far below the expected incidence. The WHO's Global Initiative for Childhood Cancer aims for a 60% survival rate by 2030, highlighting the urgency of addressing these issues. South Africa’s late diagnosis of childhood cancer is impacted by prevalent myths surrounding childhood cancer, such as misconceptions about contagion and inheritance, which hinder early diagnosis and treatment compliance. The integration of traditional health practitioners into awareness campaigns is emphasised as a vital strategy for increasing referrals and reducing stigma. The role of civil society and patient advocates in implementing educational initiatives, including the Siluan Early Warning Signs of Cancer in Children, to improve awareness among healthcare workers and communities, acts as a catalyst for progress. These initiatives, often collaborative nature, undertaken by civil society and patient advocate groups to enhance knowledge, reduce stigma and create pilot initiatives build awareness in communities and kick-start political will to develop policies that can ensure a supportive environment for children with cancer and their families.

## Background

In 2015, the International Agency for Research on Cancer (IARC) reported that the worldwide incidence of childhood cancer is increasing, from 165,000 new cases annually to 215,000 cases for children 14 years and younger and 85,000 new cases for 15–19-year-olds. Many more remain uncounted and unreported due to a lack of childhood cancer registries in many countries [1]. A 2017 report by the IARC reported that, over the past two decades, childhood cancer has increased globally by 13%.

The South African National Cancer Registry (NCR) [2] has collected information on cancer diagnoses since 1986 and the South African Children’s Tumour Registry [3] (SACTR) since 1987. Annually, in every million children globally, 150 are diagnosed with cancer [4]; in SA, we diagnose between 70 and 80 per million and half [5] of the children die because they reach the treatment centres too late [6]. According to the accepted population incidence rate, SA should diagnose around 1,963 children per year; instead, between 1,000 and 1,050 new cases are reported per year [7] – thus, currently the country is under-reporting/diagnosing childhood cancer cases by almost 95% [8].

In September 2018, the World Health Organisation (WHO) introduced the WHO Global Initiative for Childhood Cancer, [9] with the aim of reaching at least a 60% survival rate for children with cancer by 2030, thereby saving an additional 1 million lives. This new target represents a doubling of the global cure rate for children with cancer.

## The story of Khanya

On the sidelines of the 2013 Union for International Cancer Control November 2013 World Cancer Leader’s Summit in Cape Town, South Africa, the Voice of Cancer Forum – Access To Cancer Treatment In Low And Middle Income Countries – a regional perspective, Dr Barry van Emmenes told the story of Khanya’s journey to access treatment and care for a childhood cancer. Dr van Emmenes was a practising paediatrician at what was at the time the only fully dedicated and supported Paediatric Haematology Oncology Unit at Frere Hospital in the Eastern Cape, one of the nine provinces in SA.

This is his story (see video at https://vimeo.com/1111244852/a64f2945f9?share=copy):

*Based on true stories, the participants are fictitious; however, their journey, which entails a very long road they must travel to receive appropriate treatment for cancer, is very real. Khanya is an IsiXhosa word meaning light or shine and Nolumkile means wisdom.*

*As a paediatrician running this sole Paediatric Haematology/Oncology Unit in the entire Eastern Cape, situated in East London at Frere Hospital, Dr van Emmenes’ story portrays the norm rather the exception.*

*The setting of our story is in the beautiful but very remote hills of the rural Transkei. Here the locals live in mud huts, they have no electricity, no fridges, they cook all meals on an open fire. Water for drinking and washing must be collected from a river in 20l drums and carried for many kilometers on the head. For a toilet, they use the bush.*

Khanya* arrives at their local clinic walking next to his granny. His abdomen is tense and swollen and his face clearly showing his pain. It is a good thing they arrived today because the one and only sister is available to see them. Last week she was ill at home and the clinic was closed.

His gogo Nolumkile* explains to the nursing sister that Khanya has been complaining of a sore tummy for weeks now. The sister takes a look and says she’ll give him some medication for worms.

*Medicine in hand, the pair leave the clinic to wait for transport back home to their rural village. The impempe, as the locals call it, is nothing more than a 1984 Toyota “bakkie” (light truck); the odometer reads 500,000 km, but it is likely to have done far more than that. It is crudely equipped with an ill-fitting high-roofed canopy and “bankies” (benches) over the wheel arches to accommodate 10 passengers.*

*The taxi will only leave when full, so Nolumkile and Khanya must wait for more than an hour. It is expensive, costing 18US$ each way and at the end of their journey there is still a long walk up steep hills to their hut.*

*Nolumkile not only looks after Khanya, but also cares for three other grandchildren whose parents are in Johannesburg or Cape Town, seeking work.*

*She lives off her pension of 60US$ a month; with child support grants of 15US$ per month for each child, her total household income is under 125US$ a month, so she’s mindful of every dollar spent. It’s not surprising, then, that she waits a further 2 weeks before returning with Khanya to the clinic.*

*Concerned by his persistent symptoms, the clinic’s nursing sister refers the child to see a doctor at the district hospital. This necessitates a further journey for Nolumkile, which takes time and is a further burden on her limited financial resources.*

*Nolumkile is anxious about the children she has had to leave at home. A neighbour has promised to watch over them. The 10-year-old girl she’s left in charge is very responsible, but she has no cell phone, so she will be unable to find out if all is well with them.*

*The doctor at the district hospital is a community service medical officer with limited paediatric experience. However, he is able to determine that Khanya seems to have very large mass is his abdomen and therefore refers them to the Nelson Mandela Academic Hospital (NMAH) in Mthatha.*

*There is no hospital transport to the referral hospital, so yet again Nolumkile must dig into her shallow pockets to pay for private transport.*

*At NMAH Khanya is admitted to the paediatric ward, where for a week he undergoes a battery of tests which include many blood tests as well as an ultrasound and CT scan of his abdomen.*

*Finally, it is determined that he may have a tumour originating from his right kidney and the paediatric doctor at NMAH makes arrangements to transfer Khanya to Frere Hospital. They will be transported on the hospital’s ‘oncology bus’. It is a 35-seater that commutes daily between NMAH and Frere Hospital transporting patients with oncological illnesses.*

*This bus leaves the following day at 4 am. It is a 200 km journey through the Transkei and Kei Cuttings and takes a minimum of 3 hours.*

*Nolumkile has never travelled beyond Mthatha and spends most of her time worrying about the new place she is travelling to, about the fact she that her money is all spent now including the money she borrowed at 50% interest from a neighbour. Also, she has no change of clothes, only the ones that she is currently wearing. Not to mention the concern regarding Khanya’s medical illness.*

*Frere Hospital is a beacon of hope. It is the only state hospital within the Eastern Cape Province that offers comprehensive management for children with cancer, which includes access to appropriate medication, nursing and medical expertise, surgical expertise and radiotherapy, not to mention all the support received from allied health professionals.*

*I speak to Nolumkile and confirm that Khanya has indeed a large tumour arising from the right kidney, a Wilms Tumour. It is so big now that it fills most of the abdomen. There are also tumour deposits in the left kidney, as well as tumour metastases in his lungs.*

*Wilm’s tumour is a kidney cancer in children which the hospital sees quite often. But because both kidneys are involved and there are tumour metastasis in his lungs, Khanya must be flown to Cape Town for evaluation and treatment by the specialist paediatric oncology team at the Red Cross War Memorial Children’s Hospital.*

*In the Eastern Cape, facilities and resources to treat childhood cancer are scarce indeed. Looking at the rest of the country, in the Western Cape we have three hospitals (Tygerberg, Groote Schuur and Red Cross Children’s War Memorial Hospital) which offer paediatric oncology therapy, all of which are in Cape Town.*

*Gauteng has five hospitals; Free State and KwaZulu-Natal have two each. Limpopo has a shared care facility, but the Northern Cape and Mpumalanga have none.*

*Wings and Wishes, a non-governmental organisation (NGO) run by Coca-Cola, is dedicated to transporting critically ill children in order to receive lifesaving medical treatment.*

*They will cover the cost of Khanya’s flight. But they need Nolumkile’s identity document and Khanya’s birth certificate in order to book with the airline and these documents have been left at home.*

*Fortunately, there’s another mom in the ward from the same area as Nolumkile and after some calls home she is able to find someone who is travelling through to East London the following day. He commits to bring the valuable documents to Frere Hospital.*

*Delays can mean a literal difference between life and death. In Khanya’s case, if the cancer was caught early at Stage I, statistically he would have a 94.4% chance of being alive in 5 years’ time. But, in my experience, we almost never get them at this early stage. And when a child has progressed to advanced Stage IV, the survival rate drops to 54.2%.*

*Limited awareness in Primary Health Care settings account for the fact that many cases in the Eastern Cape go undiagnosed. Often those children will die in their villages or kraals, possibly in terrible discomfort or pain, without ever reaching a regional hospital.*

*After his long journey, and despite the heroic efforts of his granny, the oncology team at Frere Hospital and the Paediatric Oncologists in Cape Town, little Khanya dies.*

*He was unwell long before he ever reached us, severely underweight and malnourished. As a result of the gross poverty throughout the rural areas of the Eastern Cape many of our children are ill-equipped to face a major disease. Khanya’s little body just didn’t have the resources to cope with the illness and the treatment required.*

*There are children whose bodies do manage to withstand the onslaught of chemotherapy. But cancer treatment takes a long time and is hugely demanding of a family’s resources – up to 3 years in the case of a child diagnosed with Acute Leukaemia. In that time, it is necessary to admit the child frequently and this may be for prolonged periods of treatment.*

*I had a child last month who did not return for his second cycle of chemo for Hodgkin’s lymphoma. When we got hold of his mother, she said she had no money for transport. If she borrows 5US$ to cover the cost she will be charged 150% interest by the person who lends her the money, and they’ll keep her ID. All of this adds up to delays.*

*Originally, I did not intend that my life would be consumed by treating children with cancer – but here I am dedicating my time to it, along with a tiny cohort of staff who are stretched to breaking point, both by the lack of help and by the dearth of resources. We make it our mission to do as much as we can within our modest unit. It is tough, but we do pretty well.*

*What frustrates me most is that individuals far too quickly throw up their hands and say “I can do nothing more” because of administrative red tape.*

*Yes, no doubt we have big problems in the public healthcare system; but what kills children like Khanya is in fact a vast array of quite complex issues. If we are to fulfil the promise of improved prognosis in the treatment of childhood cancers, we have no option but to tackle each one of these challenges and solve them, remembering the promise of Section 27 of our Constitution – that everyone has the right to have access to healthcare services.*

*Poor access to treatment means that far too many of our children will die and it is my prayer that Khanya’s story must not remain a story of the norm in the treatment of children in this country with cancer.*

*November 2013, UICC World Cancer Leader’s Summit, Cape Town, South Africa*

## Perception of childhood cancer in South Africa communities

Stigma is a primary contributing reason why children affected by cancer experience late-stage diagnosis and a poorer prognosis, abscond from treatment, resulting in soaring mortality rates [10].

The LIVESTRONG Cancer Anti-Stigma Initiative was piloted in South Africa in 2010 and was implemented in 2011 with implementation partner John Snow and various South African civil society actors, including Campaigning for Cancer [11]. Activities included 11 semi-structured focus groups with key community-relevant partners in three South African townships.

In Understanding Stigma As A Barrier To Accessing Cancer Treatment In South Africa: Implications For Public Health Campaigns, Oystacher *et al* [12], reported three main labelling mechanisms: physical appearance of perceived symptoms and signs of cancer, diagnosis by a traditional health practitioner or a biomedical diagnosis by a Western physician. The authors further noted that being labelled led to anticipated discrimination, which contributed to delayed treatment; use of traditional health practitioners instead of biomedical treatment and secrecy around symptoms and/or diagnosis. Furthermore, perceptions of cancer were commonly conflated with Human Immunodeficiency Virus (HIV) and/or Tuberculosis (TB), owing to prior educational campaigns.

From published literature and the anecdotal records of its community workers and social workers, CHOC consolidated experiences of those affected by childhood cancer in communities, identifying ten prominent myths that contributed to the stigma surrounding the diagnosis and treatment of a child with cancer. These include:

## Commonly identified myths and resulting stigma in childhood cancer in South Africa

**Myth:** Cancer is contagious and can spread like flu.

**Fact:** Cancer is not contagious. Cancer cannot be spread from one child to another. We isolate children with cancer as their immunity is low and are vulnerable to infections.

**Myth:** Childhood cancers are inherited.

**Fact:** There is no known cause for most childhood cancers.

**Myth:** Children cannot get cancer.

**Fact:** The cancers that affect children are generally unique to those that affect adults. In South Africa, one in 600 hundred children is affected by cancer before the age of 16. The encouraging news is that if diagnosed early, 70%–85% of children can be cured. South Africa, less than half of the children are diagnosed early enough and reach a treatment centre in time. Many are diagnosed too late with an advanced stage of cancer for the treatment to have much chance of success and half are never diagnosed and so receive no treatment.

**Myth:** Cancer is a white-man’s disease.

**Fact:** Cancer has no respect for ethnic origin, wealth or social status. Children with cancer are fully representative of population demographics.

**Myth:** Childhood cancers are a death sentence.

**Fact:** Most childhood cancers are curable.

For example, in Acute Lymphoblastic Leukaemia, which is a common form of Leukaemia in Singapore, 3 in 4 children will be cured with chemotherapy alone. A successful cure depends on receiving the current-day standard therapy, a positive attitude and determination to overcome cancer.

**Myth**: Childhood cancer is the fault of the child or parents.

**Fact: *Childhood*** cancers are almost always caused by a DNA mutation that is not inherited but happens randomly (acquired) and *no one is at fault* for this illness.

**Myth:** There is no need for the elders to talk about cancer in the family.

**Fact:** We must talk about cancer and by talking about it, you are making other people aware of it. There is *a need* for the elders to talk about cancer in the family and decisions of transfusions or amputations can be taken *in consultation with elders* in the best interest of the child’s survival.

**Myth:** There are no symptoms and signs of childhood cancer.

**Fact:** Childhood cancer can have a range of symptoms, including, but not limited to unexplained fevers, persistent headaches, and bone pain.

**Myth:** Children do not survive cancer.

**Fact:** Most childhood cancers are curable. For example, in Acute Lymphoblastic Leukaemia, which is a common form of Leukaemia in Singapore, 3 in 4 children will be cured with chemotherapy alone. A successful cure depends on receiving the current-day standard therapy, a positive attitude and determination to overcome cancer [13].

## Methods

Advocates can play an important role in bringing evidence into the policy and health system shaping process [14]. Civil society and patient advocates that lay the groundwork for policy development through the development of community engagement initiatives, resources, tool and research. From 1999, civil society and patient advocates, individually and collaboratively, have implemented a series of interventions aimed at laying the groundwork for increased political interest and policy development for Childhood Cancer in South Africa.

### The buds of education and awareness

The South African National Department of Health has adopted and endorsed the Siluan Early Warning Signs of Cancer in Children, which the South African Association for Paediatric Haematology Oncology (SAAPHO) – previously known as the South African Children's Study Group [15] – compiled in 1999. Two-thirds of SA children with cancer never reach specialist treatment centres and the majority of those who present are in advanced stages [16].

**Figure 1.**
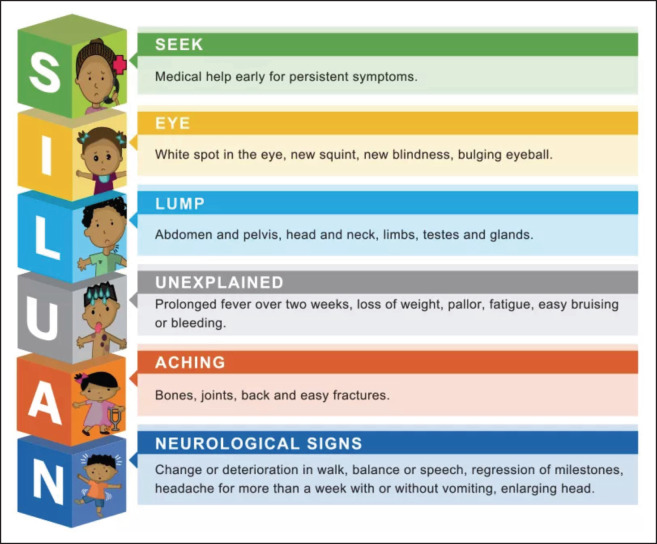
Siluan Early Warning Signs of Cancer in Children: https://choc.org.za/childhood-cancer-early-warning-signs/ [16].

Dr. Stelios Poyiadjis conducted a health promotion study to raise awareness and educate the public and primary health workers on the early warning signs of childhood cancer in Limpopo and Mpumalanga, two northern provinces in South Africa. According to the research that followed, it was found that the use of the Siluan Early Warning Signs (Figure 1) significantly increased the number of new patients referred to the Paediatric Oncology Unit at Chris Hani Baragwanath Academic Hospital in the 3–5 years following the campaign; however, it failed to generate referrals for patients at earlier stages of the disease. The findings indicated that a longer and more formal educational period for healthcare workers is needed to improve the referral of earlier stages of disease and falloffs in the number of new patients over a period, which dictates that the awareness campaign needs follow-up and on-going training. Failure to improve earlier stages of referral was surmised to be due to the time spent on the educational campaign [16].

In 2011, the Childhood Cancer Foundation SA (CHOC) embarked on a public health awareness campaign with the Gauteng Department of Health. It became evident that if the feeder areas for the paediatric oncology units, as well as healthcare workers, were not incorporated into these health campaigns, the campaigns, when initiated into a community, would not succeed in downstaging at diagnosis or in increasing the survival rate. Engagement in the communities highlighted relevant partners, who, up until this point, had not been incorporated into most cancer-related public health promotion and awareness campaigns – the traditional health practitioners.

### Creating agents of change

#### Training in early warning signs and childhood cancer

CHOC's Awareness Programme (Training and Education on the Early Warning Signs of Childhood Cancer and the debunking of myths and beliefs that lead to stigma) started in 2011. The goal of the programme is to improve early detection and facilitate effective treatment to increase the number of new diagnoses of children and teenagers with any form of childhood cancer, and to increase the survival rate of children with cancer.

Strategies employed to achieve these objectives include:

- Conducting accredited training workshops for healthcare professionals at primary healthcare clinics, public health centres and hospitals.
- Training home-based care workers, community health workers, including teachers and childminders, outreach teams and Traditional Health Practitioners on the early warning signs of childhood cancer.
- Conducting community outreach events and distributing educational material in targeted communities to address misconceptions about cancer, sharing survivor stories and running awareness campaigns.
- Highlighting issues that contribute to the lack of effective treatment outcomes for patients and advocating with decision-makers to provide solutions to such issues.
- Target audiences include healthcare professionals, healthcare workers, traditional health practitioners and communities on the early warning signs of childhood cancer.

Since its inception, the programme has trained approximately 52 00 trainees, including traditional healers, community health workers and community leaders, and reached more than 185,000 people through messaging. Giving trainees knowledge changes their attitudes towards childhood cancer, and in so doing, practices have changed and there are more referrals of possible cases to specialised treatment centres.

CHOC’s training programme has been endorsed by South Africa’s National Department of Health as well as the SAAPHO.

### Incorporating traditional healers into myths debunking

Oystacher *et al* [12], concludes that the inclusion of traditional health practitioners as key collaborators for cancer treatment should be explored to increase community member referrals, with the ultimate goal of increasing uptake of biomedical treatment [12].

In South Africa, there are 69,000 registered traditional health practitioners with the Traditional Healers Organisation. In 2007, a study by the SA Health Review found that, in total, there were 185,000 traditional health practitioners countrywide [17]. These statistics, together with each organisation’s experience in health promotion and awareness project outcomes of CHOC’s Gauteng programmes, allowed project teams to conclude that without a programme design that incorporated the training of traditional health practitioners, programmes would not reach the majority of South African children with cancer.

### Four categories of traditional health practitioners in South Africa

South Africa’s Traditional Healers Practitioners Act 22 of 2007 makes provision for four categories of traditional practitioners, all of which could play a role in the early detection of childhood cancer in South Africa.

1. A Sangoma or Diviner is initiated in healing through a cultural learning process and ceremony.
2. A Inyanga or Herbalist is not initiated but has been trained in traditional healing learned over generations, the trainers being immediate family members, such as a mother, father or grandparent, who were initiated in healing.
3. Birth Attendants known as Midwives: a traditional birth attendant or midwife, undergo formal training and assessment
4. Lingcibi, a traditional surgeon, is trained by the older generation of Lingcibi to initiate young boys to manhood. This does not only cover circumcision, but also includes vast amounts of training in taking care of the family as a man, after the father is gone, as well as other important cultural practices that happen in most African households. They help sons in all aspects of manhood.

In order to embark on incorporating these registered Traditional Health Practitioner groups, a process of consultative meetings with the leadership of the Traditional Healers Organisations in South Africa was embarked on. An outcome of these meetings included acknowledgement by the leadership of the Traditional Health Practitioners of the importance of training their members in childhood cancer and the recommendation that patient groups conducting health promotions in communities, and providing psychosocial services in treatment facilities and community structures, establish relationships with their grassroot members to understand broader issues of cultural and treatment differences between ‘Western’ and ‘Traditional’ medicine [18].

As an initial response to this recommendation, an open debate and dialogue on issues of cultural differences was held in 2015, to initiate, build and strengthen stakeholder relationships between practitioners of ‘western’ and ‘traditional’ medicine. Topics included in the dialogue agenda included death and dying, amputations, man-made disabilities and cleansing rituals, as well as the burial of man-made disabled children. At the dialogue held in 2015, the point of view put forward by the Traditional Health Practitioner group was that when their patients consulted with ‘western’ medical structures and services, they were advised to cease all treatment with traditional health practitioners. However, the role of the traditional health practitioner is broader than the physical care of the patient and extends to the cultural, spiritual, emotional and social aspects of the patient and their community [19].

The dialogue was a milestone, a breakthrough between the two practising disciplines – the way forward agreed on was to empower and build bridges between traditional and western medicine stakeholders, as currently, there was a lot for both groups to understand about the broader issues of each group. Mistrust between stakeholder groups representing both ‘western’ and ‘traditional’ medicine was identified as a major challenge. However, there appeared to be a general willingness on all sides to work together to find solutions to benefit children with cancer.

The resulting programme has to date trained 5,197 traditional health practitioners, of whom 11 are provincial leaders. Two traditional health practitioners have been incorporated into community training and health promotion efforts within the programme.

### Patient groups acting for change

Four priority actions were agreed upon at the 2011 Voice of Cancer Survivor’s National Call to Action [20], resulting from the Voice of Cancer Survivors Forum held in May 2011, a collaboration by Campaigning for Cancer and other South Africa cancer NGOs. The fourth priority of the call to action was to de-stigmatise cancer with evidence-based and culturally-relevant information that ensures the elimination of discrimination against cancer patients in their workplace and communities.

Seven years later, and after individual grassroots advocacy experiences gained in working towards this, Campaigning for Cancer and CHOC came together to develop a programme that could address childhood cancer awareness and education across SA communities. The idea began with creating the tools and resources for civil society groupings to take part in activities in a documented, co-ordinated, and evidence-based process that could be leveraged.

The programme was called VUKA KHULUMA. This is an isiZulu phrase meaning ‘to awake/arise and speak/talk’. The name embodied a powerful call to action to spread a story and talk to each other about childhood cancer.

The desired impact of the programme was to increase the survival of children and teenagers diagnosed with childhood cancer (cc) or a life-threatening blood disorder (ltbd) and to decrease disabilities relating to the late diagnosis thereof.

The teams set about developing a Memorandum of Understanding, a brand for the programme (VUKA Khuluma) and a set of change theory documents, including a Logic Frame and Implementation plan.

### Vuka Khuluma theory of change elements

Outcomes were categorised as short, medium and long-term – over a 5-year period (Table 1).

Five strategies to achieve these outcomes were defined and formed the basis for the tools and resources the programme development group would create or amend to assist in the implementation of these strategies (Table 2).

## Results

### Vuka Khuluma theory of implementation tools and resources

#### Accredited training workshops

The model of train-the-trainer was adopted in the development of five training toolkits. Each toolkit focused on a specific stakeholder grouping and their existing knowledge, and the knowledge required to enhance the health literacy of their communities about childhood cancers.

**Figure 2.**
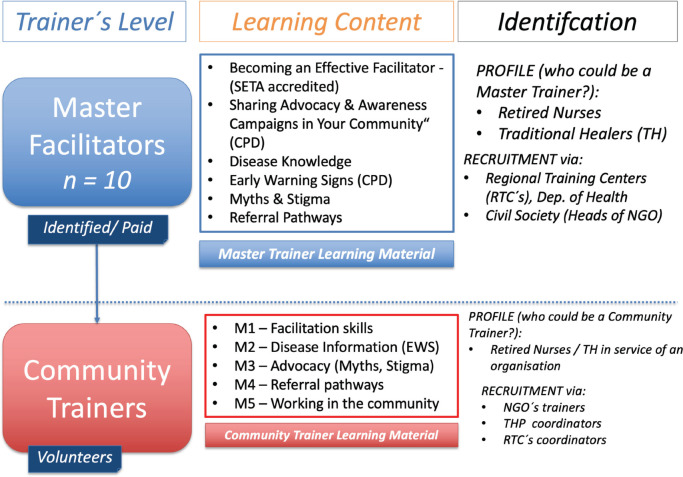
Train the trainer method of skills and knowledge differentiation amongst training models.

Trainers were categorised as Master Trainers and Community Trainers (Figure 2). Trainers were trained in advocacy theory, theory of change and facilitation skills. Master Trainers were offered the option to gain formal accreditation in the relevant modules to proficiency-related accreditation. A Master Trainer’s role was to train a cohort of volunteer Community Trainers who came from the community and would act as community information officers about childhood cancer signs and symptoms and referral pathways.

**Figure 3.**
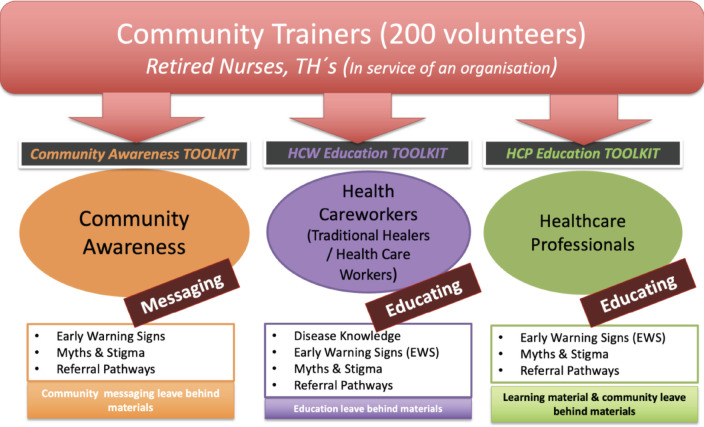
Workshops delivered by community trainers with contents aligned to target audiences.

Community Trainers would be identified amongst cohorts of HW, CHW and TH. To ensure that health literacy and literacy levels of these cohorts were considered, toolkits employed by the Community Trainer would be tailored to the outcome desired from their interaction with the community and its members, specifically considering Community Trainers who were qualified to give clinical and medical advice versus those who would provide awareness messaging to the community (Figure 3).

Each community trainer would receive a toolkit pack that would include tools, resources and templates for administrative activities to ensure adequate monitoring and evaluation data was collected as defined in the Change Theory and implementation plans, as well as material to assist the trainer during their facilitation at an education or awareness community event, including facilitation and participant guides, visual materials and community leave-behind materials that could be distributed to participants (these included pamphlets, flyer and posters to be distributed in the community). The toolkits are updated with new learnings on a continuous basis.

### Framework for baseline study of the knowledge around cancer stigma

Between 2011 and 2013, LiveSTRONG Foundation and RAND Corporation developed The Global Stigma Index toolkit as a direct outcome of the global anti-stigma project implemented in countries including SA. It seeks to empower policymakers, researchers and NGOs to assess levels of cancer stigma within a population of community [21].

The index included a 13-item list (Table 3) generating a Cancer Stigma Index Total Score that was translated into isiZulu. Answers were collected amongst community members where community awareness campaigns would be held, prior to such campaigns. A 13 question relating to childhood cancer was added to the set of questions, but not included in the calculation methodology defined by the cancer stigma index level and provided insight into identified key areas to be addressed by awareness or anti-stigma campaigns.

### Material development for community outreach awareness events and distribute educational material and Public Service Announcements campaigns across community media platforms

Existing Siluan education material was translated and formatted into various sizes for use in marketing elements, including signage poster and event advertising notices for awareness events, while community activation stands with branded materials were developed.

Toolkits with template letters in English and isiZulu, requesting permission from community leaders to allow health promotion activations, were developed. Media release templates, detailing backgrounds to the programme, facts and figures and signs and symptoms, aimed at community radio stations and publications, helped ensure that Strategy 4 activities relating to the PSA campaign reached grassroots communities and not just mainstream media in urban areas of SA.

## Conclusion

### Assets for the future

Since the implementation of the Awareness Training and Education Programme by civil society groups, it has become clear that there was a need to include childhood cancer in the Integrated Management of Childhood Illnesses (IMCI). IMCI has been developed to reduce preventable mortality, promote healthy growth and development of children under 5 years and to minimise illness and disability. A task team, including representatives from the Gauteng Department of Health, SAAPHO IMCI specialists and CHOC team members, developed an addendum for childhood cancer. Work that was informed by many of the highlighted initiatives has resulted in 2024 a pilot project initiated in Gauteng, training professional nurses on how to use the principles of IMCI to identify possible childhood cancer cases, and how to refer the patients urgently. With the buy-in of the National Department of Health and the roll-out of the programme nationally, this will help to decrease the mortality and morbidity of children with cancer. The result will be to increase the survival rate and assist in reaching the WHO Global Initiative for Childhood Cancer 2030 goal of 60% survival globally, thereby saving an additional one million lives [22]. This new target presents a doubling of the global cure rate for children and teenagers with cancer.

The NGO sector in SA collectively advocates for the cause of childhood cancer so as to influence decision-makers (nationally and internationally) to ensure comprehensive and adequate care for all children and teenagers with cancer or life-threatening blood disorders. In South Africa, it is a constitutional right for every child to have access to specialised treatment, essential medicine and supportive care. Working together to implement smaller initiatives, relevant stakeholders in the medical community and community leaders, patient advocates and civil society groups, as well as with alliances and networks, provides a basis showcase successes that can create awareness in communities and political will.

## Conflicts of interest

Lauren Pretorius is an employee of Campaigning for Cancer and director of World Bladder Cancer Patient Coalition. Adri Ludick is an employee of CHOC Childhood Cancer Foundation.

## Author contributions

Both authors contributed equally to the content.

## Figures and Tables

**Table 1.** Theory of change outcomes for Vuka Khuluma Programme.

Outcomes		
**Short term year 1 and 2 (Y1&Y2)**	**Medium term year 3 and 5 (Y3 & Y4)**	**Long term year 5**
• By Y2, increase the awareness among parents and caregivers of cc and relating stigmas and knowledge of early warning signs and benefits of treatment compliance. • By Y2, educate the community on EWS (Early Warning Signs) and myths relating to cc and ltbd. • By Y2, increase the number healthcare workers, community healthcare workers (CHW) and traditional health practitioners (TH) educated in knowledge and attitudes relating to cc and ltbd in the targeted area. • By 2019, document all gaps/barriers in existing supporting services and referral pathways that impact treatment compliance.	• By Y3, increase the awareness amongst targeted communities about their role of supporting families with children diagnosed with cc. • By Y3, create awareness and educate 95% of HCP, CHW and TH of existing supportive services and referral pathways. • By Y4, reduce community stigma of cc and ltbd in the targeted area. • By Y4, ensure influence in the improvement of supportive services and referral pathways amongst decision makers, resulting in 50% of diagnosed children being treatment-compliant.	• By Y5 increase the number of children diagnosed with cancer and ltbd to 130 / million in the area. • By Y5, increase the number of children diagnosed with CC in stage 1 and 2, to 60% of children diagnosed the target area. • By Y5, ensure access to appropriate treatment and the continuation and compliance thereof to 80% of those children diagnosed in the area.

**Table 2.** Vuka Khuluma Programme theory of change strategies.

Strategies
Conduct accredited training workshops for healthcare professionals and non-professionals (traditional healers and community healthcare workers) working in clinics, hospitals and healthcare/childhood civil society locations.
Conduct a baseline study of the knowledge on cancer stigma by collecting in-depth data on the public’s knowledge, attitudes, and health practices regarding childhood cancer and stigma in targeted communities.
Conduct community outreach awareness events and distribute educational material in targeted communities to message misconceptions about cancer; share survivor stories and awareness campaigns.
Conduct public service announcements campaigns across community media platforms to raise awareness and engage community about cc and cc-related myths.
Highlight issues that contribute to lack of good treatment outcomes for patients, and advocate with decision makers to provide solutions to such issues.

**Table 3.** Cancer Stigma Index score questionnaire.

#	Question	Not at all	A little bit	Some- what	Quite a bit	Very much
1	I would feel uncomfortable talking to a person with cancer					
2	Treatment and support are useless for someone with cancer					
3	I would feel uncomfortable sitting next to someone with cancer					
4	I would feel uncomfortable sending my own child to school with another child with cancer					
5	I would feel uncomfortable if someone with cancer lived nearby me					
6	If a close friend had cancer, I would avoid him/her					
7	I would feel uncomfortable being friends with someone with cancer					
8	People can only blame themselves for getting cancer					
9	If I had cancer, I would be ashamed of myself					
10	I would feel isolated/alone if I received treatment for cancer					
11	If my child had cancer, I would be ashamed of him/her					
12	If my child had cancer, I would consider leaving him/her					
13	When I hear that a child has cancer I automatically think that he/she is going to die					

Adapted from LIVESTRONG FOUNDATION Global Stigma Index
